# Associations of plasma fibroblast growth factor 23 and other markers of chronic kidney disease—Mineral and bone disorder with all-cause mortality in South African patients on maintenance dialysis: A 3-year prospective cohort study

**DOI:** 10.1371/journal.pone.0216656

**Published:** 2019-05-20

**Authors:** Bala Waziri, Eustasius Musenge, Raquel Duarte, Caroline Dickens, Therese Dix-Peek, Vakhtang Rekhviashvili, Graham Paget, Saraladevi Naicker

**Affiliations:** 1 School of Public Health, University of the Witwatersrand, Johannesburg, South Africa; 2 Department of Internal Medicine, Faculty of Health Sciences, University of the Witwatersrand, Johannesburg, South Africa; 3 Department of Medicine, Ibrahim Badamasi Babangida Specialist Hospital, Minna, Nigeria; 4 Renal Unit, Donald Gordon Medical Center, University of the Witwatersrand, Johannesburg, South Africa; Purdue University, UNITED STATES

## Abstract

**Introduction:**

Few studies have linked high levels of plasma C-terminal fibroblast growth factor 23 (FGF23) with poor clinical outcomes in patients on maintenance haemodialysis (MHD), while the association between intact FGF23 and mortality in this group of patients remains inconclusive.

Therefore, the aim of this study was to evaluate the association between plasma levels of intact FGF23 and mortality in dialysis patients.

**Methods:**

A prospective multicenter study involving patients undergoing dialysis at three dialysis centers in Johannesburg was undertaken between 1^st^ October 2014 and 31^st^ December 2017.

**Results:**

The study comprised 165 chronic dialysis patients (111 blacks, 54 whites) with a mean age of 46.6 ±14.2 years. During a three year follow up period, there were 46 deaths (1.03 per 100 person-years). The median plasma FGF 23 level was 382 pg/ml (interquartile range [IQR], 145–2977). In adjusted multivariable analyses, there was a non-statistically significant increase in the risk of mortality with higher quartiles of FGF 23 levels: the adjusted hazard ratios (HR) for the second, third and fourth quantiles were HR 3.20 (95% CI, 0.99–10.35; P = 0.052), HR 2.43(95% CI,0.65–9.09; P = 0.19), and HR 2.09 (95% CI, 0.66–7.32; P = 0.25),respectively. Corrected serum calcium 2.38–2.5 mmol/l [HR 2.98 (95% CI, 1.07–8.29; P = 0.04] and > 2.50 mmol/l [HR 5.50 (95% CI, 1.84–16.48; P = 0.002] were independently associated with increased risk of death. Likewise, patients with intact parathyroid hormone > 600 pg/ml had a 3.46-fold higher risk of death (HR 3.46, 95% CI, 1.22–9.82 P = 0.019). These findings persisted in time -dependent analyses.

**Conclusion:**

Higher levels of intact FGF 23 appear not to be independently associated with all-cause mortality in our dialysis patients, while hypercalcaemia and severe hyperparathyroidism were found to be independent predictors of mortality in this cohort of patients.

## Introduction

Since the discovery of fibroblast blast growth factor 23(FGF23) in 2000[1], this novel phosphaturic biomarker has gained global recognition and continues to be associated with several adverse clinical outcomes[2–4]. Likewise, the discovery of FGF23 has led to further insights into the pathogenesis of chronic kidney disease–mineral and bone disorder (CKD-MBD), accounting for the paradigm shift from the classic trade off hypothesis to an updated trade off hypothesis[5].

The incidence of mortality remains unacceptably high in CKD patients, despite interventions on traditional risk factors and thus the need to identify other non-traditional risk factors for death in CKD patients, such as FGF23. Additionally, cardiovascular disease (CVD) is one of the leading causes of death in CKD populations, with CKD-MBD identified as an emerging entity responsible for the increased risk of CVD[6, 7]. Hence, since FGF 23 is one of the main regulators of the CKD-MBD axis, it became necessary to investigate its role as a risk factor for mortality in CKD populations.

In 2008, Gutierrez et al. were the first to report a significant association between higher levels of FGF23 and mortality in a haemodialysis population. In this large US study, involving incident haemodialysis patients, a monotonic dose–response relationship between FGF23 levels and mortality was reported; patients in the highest quartile of FGF23 had increased risk of death compared to patients in the lowest quartile[2]. However, these findings were not consistent nor replicated in all the subsequent studies, with some of the studies revealing a non-significant association between FGF23 and mortality[8, 9]. The observed discrepancy in these studies was partly attributed to differences in assay methodology and whether intact or C terminal FGF 23 was utilized. The C- terminal enzyme-linked immunosorbent assay (ELISA) can recognize both the full length FGF23 and the C-terminal fragments obtained from FGF23 proteolysis. While the intact FGF23 assay detects the full length FGF23 molecule[10]. The results obtained by both assays methodology varied and thus lack of harmonization of these assays make it difficult to make comparisons across different studies. The intriguing complexity of assay methodology is further compounded by racial variations in the levels of FGF23 as reported in prior studies[11, 12]. For example, in a recent meta-analysis, a sub analysis based on race, FGF 23 assay type and dialysis modality showed variations in the association between elevated FGF-23 and mortality in haemodialysis patients[13]. The pathophysiological explanation behind the reported association between FGF23 and mortality remains unclear. Some of the proposed mechanisms include a possible direct cytotoxic effect of excess FGF23 on the myocardium leading to left ventricular hypertrophy and arterial calcification through phosphorus and vitamin D regulation[3, 14, 15]. Similarly, since the regulation of CKD-MBD by FGF23 is through an interplay with other known traditional markers of CKD-MBD such as calcium, phosphate and PTH, this current study will further explore the relationships between these markers of CKD-MBD and mortality.

Furthermore, studies largely from Europe, America and Asia have also linked calcium, parathyroid hormone, phosphate to increased risk of mortality in patients undergoing dialysis[16, 17]. While in Africa, the risk factors of mortality in patients undergoing haemodialysis are yet to be fully explored, with most studies focusing on traditional risk factors such as diabetes mellitus, hypertension, dyslipidaemia and anaemia[18, 19]. To the best of our knowledge, no studies have evaluated the association between FGF23 and mortality in African patients on dialysis. Therefore, this study aimed to determine the relationship between FGF23, traditional markers of CKD-MBD and mortality in patients on dialysis; and tested the hypothesis that high level of plasma FGF23 is expected to be an independent predictor of all -cause mortality in South African patients on maintenance haemodialysis.

## Materials and methods

This was a prospective multicenter study involving patients undergoing chronic haemodialysis and peritoneal dialysis at three dialysis centers in Johannesburg between October 2014 and December, 2017. Eligible patients were aged 18 years and above with established end stage kidney disease (ESKD) on dialysis and have no active malignancy or a history of parathyroidectomy. Patients with acute kidney injury and non-consenting to participate were excluded.

Information obtained included participants‘ socio-demographic characteristics, history of comorbid conditions as diabetes mellitus, usage of CKD-MBD medications, aetiology of end stage kidney disease (ESKD) and blood pressure measurements. Determination of race was based on self-report by the participants.

### Laboratory measurement

Detailed blood sample collections and test methodologies as previously published[12], are briefly described below:

Blood samples were collected at baseline for measurements of FGF23 and other biochemical markers of CKD-MBD. Plasma PTH, serum calcium and phosphate were repeatedly measured at intervals of three months. Results of six months post enrollment into the study were used for time varying analyses.

“Plasma FGF23 was measured using a sandwich-based enzyme-linked immunosorbent assay kit from EMD Millipore Corporation (Billerica, MA, USA); lower limit of detection was 3.2 pg/ml. The intra and inter assay coefficients of variation (CVs) were < 11%.

Plasma intact PTH was measured by an electrochemiluminescence immunoassay (ECLIA) run on a Cobas 6000 auto analyzer (Roche Diagnostics, Mannheim, Germany). The intra and inter assay coefficients of variation (CVs) were <2% and <3.4%, respectively.

Plasma 25 hydroxyvitamin D (25-OHD) was measured using the high performance liquid chromatography (HPLC) kit (Recipe, Munich, Germany). The intra and inter assay coefficients of variation (CVs) were < 5%. Our institutional laboratory is a participating member in the vitamin D external quality assurance scheme (DEQAS).

Serum calcium, phosphate and alkaline phosphatase were measured using the ADVIA 1800 centaur auto analyzer; Siemens Diagnostics, Tarrytown, USA” (11).

#### Primary outcome and exposure variables

The primary endpoint was all-cause mortality, events other than this endpoint such as kidney transplantation, loss to follow up and still on dialysis at end of the study were censored. The primary exposure variable of interest was intact FGF23. Secondary exposure variables were traditional markers of CKD-MBD (phosphate, calcium, PTH, bone specific alkaline phosphatase, and 25-OHD).

In line with previous studies[2, 20], plasma FGF23 was categorized into quantiles based on 25, 50 and 75 percentile distributions and using levels lower than the 25 percentile as the reference value.

FGF23 values lower than 25 percentile were considered low and values above 50 percentile as high. Similarly, due to the lack of recommendations for clinical cut-off values for bone specific alkaline phosphatase (BSALP) and in line with a previous study[21], participants were categorized based on 25, 50 and 75 percentile distributions and the interquartile range was taken as the reference value. The use of the interquartile range as the reference point was in line with a previous study that has linked both low and high levels of total alkaline phosphatase to mortality[22], therefore, we hypothesized that low and high BSALP may also be associated with all–cause mortality. Furthermore, in the absence of established cut off values for BSALP, the use of median value in dichotomizing participants into low versus high BSALP as adopted by some previous studies[23] may preclude detecting a nonlinear relationship between BSALP and mortality in these patients. Hence, the rationale for adopting an interquartile range as the reference category in this study.

Plasma Intact PTH, serum calcium and phosphate were categorized based on the Kidney Disease Improving Global Outcomes (KDIGO) recommended target values and in line with a previous study[16, 24]. Briefly, the KDIGO guidelines recommend maintaining intact PTH in the range of two to nine times the upper normal limit for the assay, while serum calcium and phosphate should be maintained within the normal laboratory reference values. Hence, the normal laboratory references for serum calcium and phosphate which were almost the same with their interquartile ranges were used as the reference values. Serum calcium was categorized into five categories based on < 10^th^ percentile (%), 10–25%, 25–75%, 75–95% and >95% distributions.

The exposure variables of interest were further modelled on a continuous scale.

Model 1 adjusted for age, calcium, phosphate, Bone Specific alkaline phosphate_,_ intact PTH and 25-hydroxyvitamin D. Model 2 in addition to variables in model 1, further adjusted for race, gender, diabetes status, use of calcium carbonate, alfacalcidol, dialysis modality and vintage.

### Ethical consideration

The research protocol was approved by the Health Research and Ethics Committee of the University of the Witwatersrand; clearance certificate number M141016. Written informed consent was obtained from each patient before enrollment into the study.

### Statistical analysis

Descriptive statistics were used to characterize study participants. Comparison of baseline parameters between patients that died and survived was done using chi square, unpaired t tests and Mann- Whitney tests for categorical outcomes, and normally distributed and non-normally distributed continuous variables respectively. We also used one way ANOVA and Kruskal -Wallis tests to compare participants’ parameters across quartiles of FGF23, PTH and calcium. A Cox regression model was initially fitted with baseline fixed variables (calcium, phosphate and PTH) and then repeated as time varying covariates. Eligibility of variables into the Cox multivariable regression model was determined using a stepwise regression strategy; a backward elimination method was used to include variables with specified p values less than 0.20 into the model. Variables that are known to be plausibly associated with death in CKD were assessed for eligibility and forced into the model even if their p values were >0.2. Plasma FGF23 and PTH were log transformed and then introduced along with other exposure variables into the Cox model on a continuous scale. The nonlinear association between the explanatory variables and all-cause mortality was explored using a fractional polynomial. The proportional hazards assumption was checked using Schoenfeld residuals test. The Schoenfeld global test chi square was 18.96 with a p value of 0.65 and the individual p values for the fitted Cox models were >0.05. Hence, we failed to reject the null hypothesis that states no violation of proportional-hazards assumption.

We further explored whether race modified the effects of our primary exposure variable on all-cause mortality by adding interaction terms of race with categories of FGF23 into the model. This model was compared to model 2 (fully adjusted model) using the likelihood-ratio test.

Model 1 adjusted for age, calcium, phosphate, Bone Specific alkaline phosphate_,_ intact PTH and 25-hydroxyvitamin D.

Model 2 in addition to variables in model 1, further adjusted for race, gender, diabetes status, use of calcium carbonate, alfacalcidol, dialysis modality and vintage.

All analyses were performed using STATA version 12 (STATA Corp., TX, USA).

## Results

The study comprised 165 dialysis patients (111 blacks, 54 whites) with a mean age of 46.5 ±14.2 years. Most of the study participants were on CKD–MBD related medications: calcium carbonate (66. 7%) and Alfacalcidol (63.0%) (Table 1).

**Table 1:** Participants’ characteristics by categories of plasma Fibroblast growth factor 23.

Variables	All(N = 165)	Intact FGF23 categories (pg/ml)				p -value
		<148 (n = 42)	148–382 (n = 40)	383–2977 (n = 41)	>2977 (n = 42)	
Age (years)	46.6±14.2	46.2±13.9	49.6±15.7	47.7±14.2	42.7±11.6	0.16
Gender n(%)						
Male	90 (54.5)	22(52.4)	19(47.5)	23 (56.1)	26 (61.9)	0.39
Female	75(45.5)	20(47.6)	21(52.5)	18(43.9)	16 (38.1)	
Race n(%)						
Black	111(67.3)	32(76.2)	27 (67.5)	28 (68.3)	24 (57.1)	0.18
White	54(32.7)	10(23.8)	13 (32.5)	13 (31.7)	18 (42.9)	
Dialysis Vintage(months)	61(43–96)	59(42–91)	59(39–108)	68(42–102)	62(48–85)	0.91
DM n(%)	13 (7.9)	3 (7.1)	2 (5.0)	3 (7.3)	4 (9.5)	0.83
Systolic BP(mmHg)	143±26	144±27	141±22	145±28	143±26	0.91
Diastolic BP (mmHg)	86±23	93±30	83±19	83±21	86±16	0.25
Hb (g/dl)	10.8±2.1	10.8±2.4	11.2±2.0	10.5±1.8	10.5±2.4	0.39
T.cholesterol (mmol/l)	4.30±1.43	4.26±0.95	4.20±1.35	4.20±1.41	4.53±1.96	0.80
Calcium (mmol/l)	2.21±0.28	2.14±0.27	2.14±0.29	2.27±0.15	2.27±0.33	0.03
IPTH(pg/ml)	750(268–1359)	534(226–1193)	694(213–1034)	784(312–1736)	888 (406–1560)	0.11
Phosphate (mmol/l)	1.52±0.55	1.23±0.34	1.39±0.51	1.58±0.52	1.89±0.60	<0.001
BSALP (IU/L)	15.8±5.5	16.4±5.4	15.6±5.0	15.3±4.7	15.6±6.2	0.87
Albumin (g/dl)		36.3±6.9	37.2±4.7	35.7±5.3	35.9±7.1	0.74
25-OHD (ng/ml)	27.7±13.6	27.6±15.1	24.5±11.9	31.9±14.2	26.0±13.5	0.09
Alfacalcidol n(%)	104 (63.0)	24(57.1)	25(62.5)	28(68.3)	27(64.3)	0.30
CaCo_3_ n(%)	110(66.7)	26(61.9)	28(70.0)	26(63.4)	30(71.4)	0.47

T = Total; BP = Blood pressure; FGF23 = Fibroblast growth factor; BSALP = Bone specific alkaline phosphatase; PTH = Parathyroid hormone, 25-OHD = 25 -hydroxyvitamin D; CKD-MBD-Chronic kidney disease- mineral bone disease; Caco_3_ = Calcium carbonate, continuous variables are reported as means± standard deviations or medians(interquartile ranges)

The mean serum phosphate was higher in patients within the highest quartile of FGF23 and this increased in ascending order across the categories. Other baseline parameters were comparable across the categories of FGF23 (Table 1).

As shown in the supplementary S1 Table, Patients with PTH within KDIGO recommended target values tend to be older.

Patients with serum calcium within the range of 2.38–2.51 mmol/l had significantly higher levels of plasma PTH. Other characteristics were comparable across the categories of serum calcium (S2 Table).

During a follow up period of 3 years with a mean time of 27±9 months, 46 deaths (1.03 per 100 person- years) occurred, 11 (6.7%) patients had a successful kidney transplant and only 2 (1.2%) patients were lost to follow-up.

The baseline clinical characteristics of the study participants by race are shown in Table 2.

**Table 2:** Study participants characteristics by race.

Variable	All(N = 165)	Black(n = 111)	White(n = 54)	p -value
Age (years)	46.6±14.2	42.8±11.5	54.4±16.0	<0.001
Outcome n (%)				
Dead	46 (27.9)	27 (24.3)	19 (35.2)	0.14
Alive	119 (72.1)	84 (75.7)	35(64.8)	
SBP (mmHg)	143±26	142±24	146±29	0.47
Hb (g/dl)	10.8±2.1	10.7±2.2	11.0±2.0	0.32
T. Cholesterol (mmol/l)	4.30±1.43	4.28±1.44	4.36±1.43	0.79
Calcium (mmol/l)	2.21±0.28	2.18±0.31	2.27±0.21	0.06
Albumin (g/L)	36.1±6.7	36.6±6.8	35.8±4.1	0.51
Phosphate (mmol/l)	1.52±0.55	1.44±0.57	1.72±0.45	0.003
BSALP (U/L)	15.8±5.3	16.1±5.7	15.0±4.2	0.19
PTH (Pg/ml)	750 (268–1359)	856(314–1636)	440 (161–890)	0.003
25-OH vit D (ng/ml)	27.7±13.6	29.9±13.7	22.3±12.6	<0.001
FGF 23 (pg/mL)	382 (145–2977)	322 (105–2543)	970 (187–3777)	0.01
CKD-MBD med n(%)				
Alfacalcidol	104(63.0)	74(66.7)	30(55.5)	0.41
Calcium carbonate	110(66.7)	77(69.3)	33 (61.1)	0.13

T = Total; SBP = Systolic Blood pressure; Hb = Haemoglobin; FGF23 = Fibroblast growth factor; BSALP = Bone specific alkaline phosphatase; PTH = Parathyroid hormone, 25-OHD = 25 -hydroxyvitamin D; CKD-MBD-Chronic kidney disease- mineral bone disease.

In comparison to Whites, Blacks were significantly younger with higher median intact PTH, mean 25-OHD and lower median plasma FGF 23 levels.

### FGF23 and mortality

In comparison to patients in the lowest quartile of FGF23, patients in the second, third and fourth quartiles had non-statistically significant increased unadjusted hazard ratios (HR) in the univariable regression analysis. Although the HR became accentuated in the multivariable Cox analysis, it still failed to reach statistical significance. The fully adjusted hazard ratios (HR) for the second, third and fourth quartiles were HR 3.20 (95% CI, 0.99–10.35; P = 0.052), 2.43 (95% CI,2.43(0.65–9.09; P = 0.19, and HR 2.09 (95% CI, 0.66–7.32; P = 0.25), respectively (Table 3, model 2).

**Table 3:** Fixed and time varying hazard ratios for all-cause mortality.

	Fixed Hazard ratios for all- cause mortality					
Variable	Unadjusted HR(95 CI)	P-value	Adjusted HR (95%CI) Model 1	P-value	Adjusted HR(95 CI) Model 2	p-value
FGF 23						
<148	1.00 (reference)		1.00(reference)		1.00(reference)	
148–382	1.80 (0.76–4.23)	0.18	2.31(0.87–6.08)	0.09	3.20(0.99–10.35)	0.052
383–2977	1.49 (0.63–3.48)	0.36	1.11(0.41–3.02)	0.83	2.43(0.65–9.09)	0.19
>2977	1.28 (0.52–3.15)	0.60	1.11(0.39–3.12)	0.85	2.09(0.66–7.32)	0.25
Calcium						
<1.80	1.45 (0.49–4.27)	0.50	1.68(0.51–5.50)	0.39	1.08(0.20–5.35)	0.93
1.80–2.10	0.50 (0.15–1.69)	0.27	0.75(0.21–2.71)	0.66	0.98(0.25–3.77)	0.98
2.11–2.37	1.00 (reference)		1.00(reference)		1.00(reference)	
2.38–2.50	2.21(1.05–4.66)	0.04	3.32(1.35–8.15)	0.009	2.98(1.07–8.29)	0.04
>2.50	2.94(1.17–7.40)	0.02	5.14(1.88–14.03)	0.001	5.50(1.84–16.48)	0.002
PTH						
<130	1.03(0.22–4.87)	0.97	1.05(0.30–3.73)	0.94	1.15(0.29–4.63)	0.96
≥130-≤600	1.00		1.00(reference)		1.00(reference)	
>600	2.23(1.03–4.85)	0.04	2.77(1.09–7.08)	0.03	3.46(1.22–9.82)	0.019
Phosphate						
<1.15	0.80(0.35–1.81)	0.59	1.50(0.57–3.91)	0.42	2.76(0.93–8.19)	0.07
1.14–1.75	1.00		1.00(reference)		1.00(reference)	
>1.75	1.41(0.72–2.78)	0.32	2.35(1.09–5.06)	0.03	1.64(0.60–4.51)	0.33
BSALP						
<12	1.87(0.68–5.14)	0.23	3.20(0.99–10.28)	0.051	1.64(0.44–6.12)	0.46
12–15	1.00(reference)		1.00(reference)		1.00(reference)	
>15	2.37(0.98–5.71)	0.055	2.91(1.12–7.54)	0.028	2.12(0.77–5.87)	0.15
Time Dependent Hazard ratios						
FGF 23						
<148	1.00(reference)		1.00(reference)		1.00(reference)	
148–382	1.80(0.76–4.23)	0.18	2.49(0.99–6.21)	0.051	2.61 (0.68–9.91)	0.16
383–2977	1.49(0.63–3.48)	0.36	1.54(0.61–3.89)	0.36	1.89(0.48–9.91)	0.36
>2977	1.28(0.52–3.15)	0.60	1.48(0.57–3.84)	0.42	2.06(0.64–6.57)	0.69
Calcium						
<1.80	2.02(0.69–5.92)	0.20	2.34(0.72–7.64)	0.16	2.56 (0.63–10.25)	0.18
1.80–2.10	0.80(0.33–2.09)	0.71	1.19(0.45–3.88)	0.72	0.96 (0.31–2.94)	0.94
2.11–2.37	1.00(reference)		1.00(reference)		1.00 (reference)	
2.38–2.50	1.70(0.72–4.02)	0.23	2.03(0.72–5.74)	0.18	1.62 (0.51–5.22)	0.46
>2.50	4.54(2.05–10.05)	<0.001	4.59(1.82–11.62)	0.001	6.44 (2.24–18.49)	0.001
PTH						
<130	1.02(0.12–8.29)	0.99	1.73(0.41–7.25)	0.45	1.15(0.23–5.69)	0.87
≥130-≤600	1.00(reference)		1.00(reference)		1.00(reference)	
>600	3.02(1.34–6.82)	0.008	3.51(1.36–9.07)	0.009	3.75(1.39–10.07)	0.009
Phosphate						
<1.15	0.81(0.38–1.72)	0.58	1.54(0.64–3.68)	0.33	2.13 (0.56–8.16)	0.26
1.15–1.75	1.00(reference)		1.00(reference)		1.00 (reference)	
>1.75	1.15(0.59–2.22)	0.68	1.35(0.65–2.83)	0.43	1.30 (0.42–3.99)	0.65
BSALP						
<12	1.87(0.68–5.14)	0.23	2.47(0.81–7.53)	0.11	1.23(0.35–4.43)	0.72
12–15	1.00(reference)		1.00(reference)		1.00(reference)	
>15	2.37(0.98–5.71)	0.055	2.61(1.03–6.34)	0.04	1.92(0.69–5.23)	0.20

HR = Hazard ratio, PTH = Parathyroid hormone, BSALP = Bone Specific Alkaline Phosphate, FGF23 = Fibroblast growth factor 23

Model 1 adjusted for age, calcium, phosphate, Bone Specific alkaline phosphate, intact PTH and 25-hydroxyvitamin D.

Model 2 in addition to variables in model 1, further adjusted for race, gender, diabetes status, use of calcium carbonate, alfacalcidol, dialysis modality and vintage. A marker of mineral bone disease is not included in the multivariable analysis whenever it is being considered as an explanatory variable.

Since categorizing explanatory variables may be associated with loss of information, further examination of FGF23 on a continuous scale did not reveal a statistical significant association at both univariable (HR per unit increase in natural logFGF23, 1.04; 95% CI, 0.87–1.25, P = 0.86) and multivariable Cox regression analyses (HR per unit increase in natural logFGF23, 1.02; 95% CI, 0.81–1.29, P = 0.54)(Table 4).

**Table 4:** Hazard ratios for all-cause mortality factoring explanatory variables on a continuous scale.

Variable	Univariable	p- value	Adjusted HR (95%CI) model 1	p -value	Adjusted HR (95%CI) Model 2	p -value
Log FGF23	1.04(0.87–1.25)	0.86	0.90 (0.71–1.14)	0.38	1.02 (0.81–1.29)	0.86
Log PTH	2.26(1.26–4.04)	0.006	3.13 (1.54–6.37)	0.002	2.21 (1.07–4.55)	0.03
Calcium	3.01(1.005–9.61)	0.049	5.13 (1.24–21.09)	0.02	5.34 (1.11–25.73)	0.04
Phosphate	1.06(0.96–1.18)	0.24	2.27 (1.11–4.64)	0.03	2.12 (1.02–4.42)	0.045
BSALP	1.01(0.96–1.07)	0.62	1.02 (0.97–1.09)	0.38	1.03 (0.96–1.10)	0.41

FGF23 = Fibroblast growth factor 23, BSALP = Bone Specific Alkaline Phosphatase, CI = Confidence interval, HR = Hazard ratios

Model 1 adjusted for age, calcium, phosphate, Bone Specific alkaline phosphate, intact PTH and 25-hydroxyvitamin D.

Model 2 in addition to variables in model 1, further adjusted for race, gender, diabetes status, use of calcium carbonate, alfacalcidol and dialysis modality. A marker of mineral bone disease is not included in the multivariable analyses whenever it is being considered as an explanatory variable.

**Fig 1:**
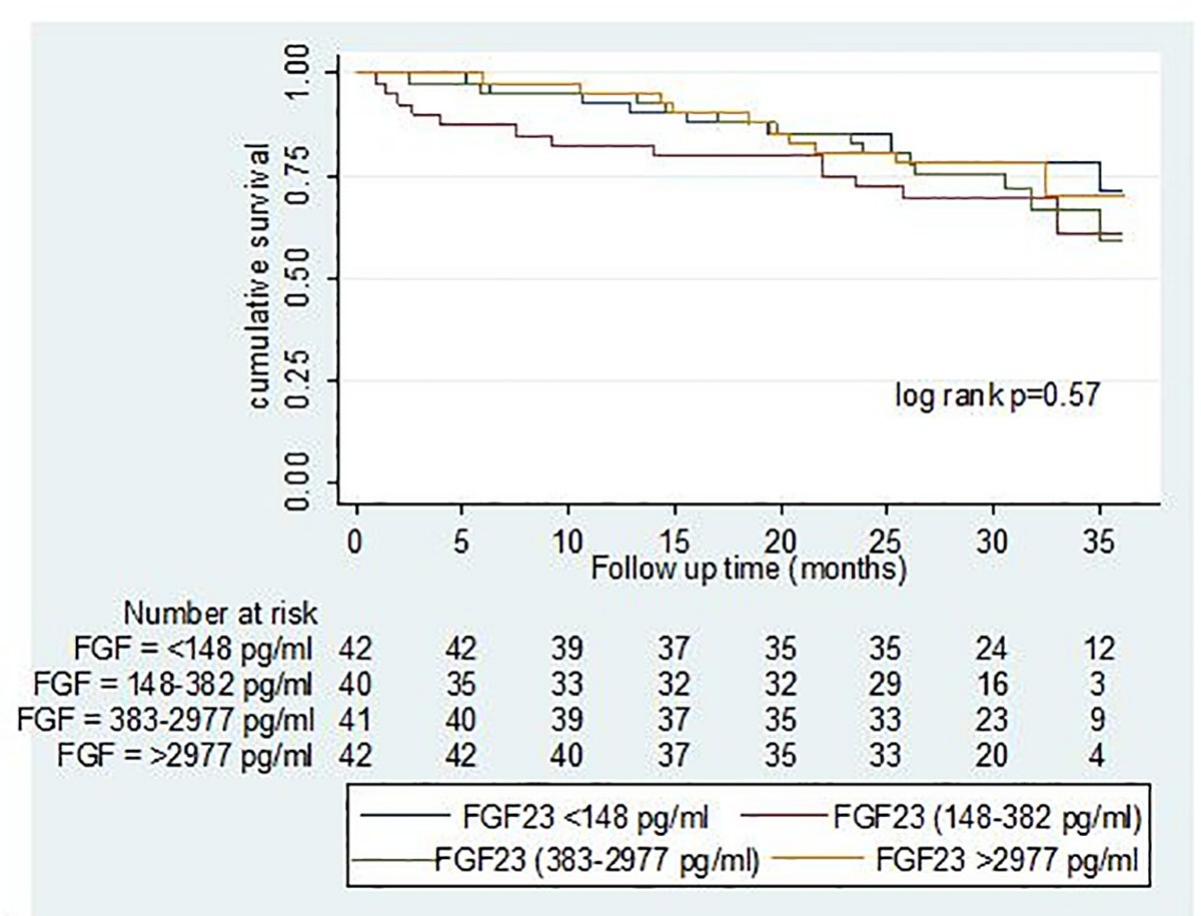
Kaplan Meier survival curves for different categories of plasma Fibroblast growth factor 23. FGF = Fibroblast growth factor 23.

Fig 1 represents the Kaplan Meir survival curves by different categories of FGF23; there is no statistically significant difference in the survival functions across the categories (log rank p >0.05).

### Calcium and mortality

**Fig 2:**
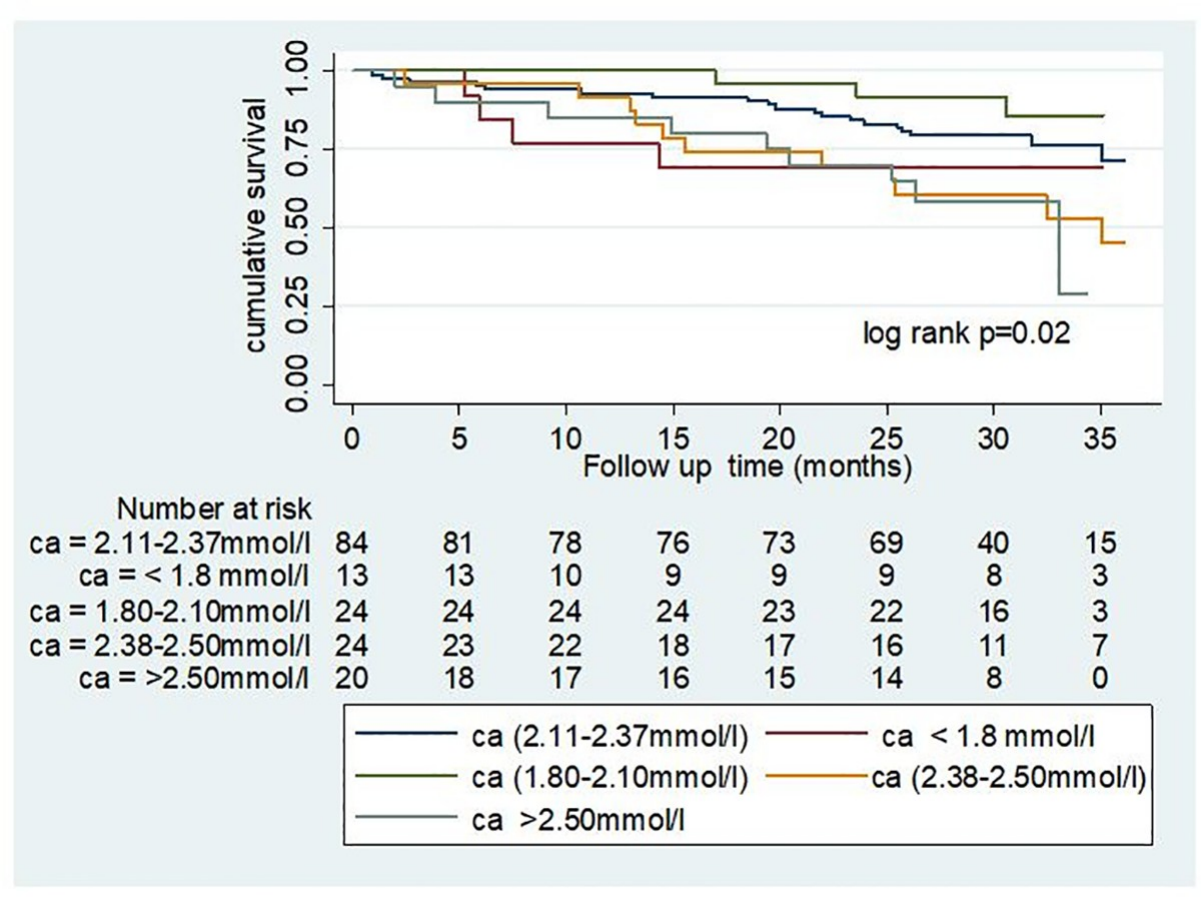
Kaplan Meier survival curves for different categories of serum calcium; ca = calcium.

Participants whose calcium levels were outside the laboratory normal range had increased risk of death. The HRs for baseline serum calcium values 2.38–2.5 mmol/l and > 2.5 mmol/l remained persistently high and significant throughout the models (1 and 2): the final model 2 adjusted HR for serum calcium 2.38–2.5 mmol and > 2.50 mmol were HR 2.98 (95% CI, 1.07–8.29; P = 0.04) and HR 5.50 (95% CI, 1.84–16.48; P = 0.002), respectively (Table 3). Fig 2 represents the Kaplan Meier survival curves by different categories of serum calcium; patients with serum calcium ≥2.38 mmol has worst survival function.

### Intact PTH and mortality

In the fully adjusted Cox regression model 2, participants with intact PTH above the upper limit of the KDIGO recommended target value versus those within the target values had a 3.46 fold higher risk of death (HR 3.46, 95% CI, 1.22–9.82 P = 0.019).This relationship persisted when PTH was considered as a time varying co-variate (HR 3.75, 95% CI, 1.39–10.07, P = 0.009 (Table 3).

**Fig 3:**
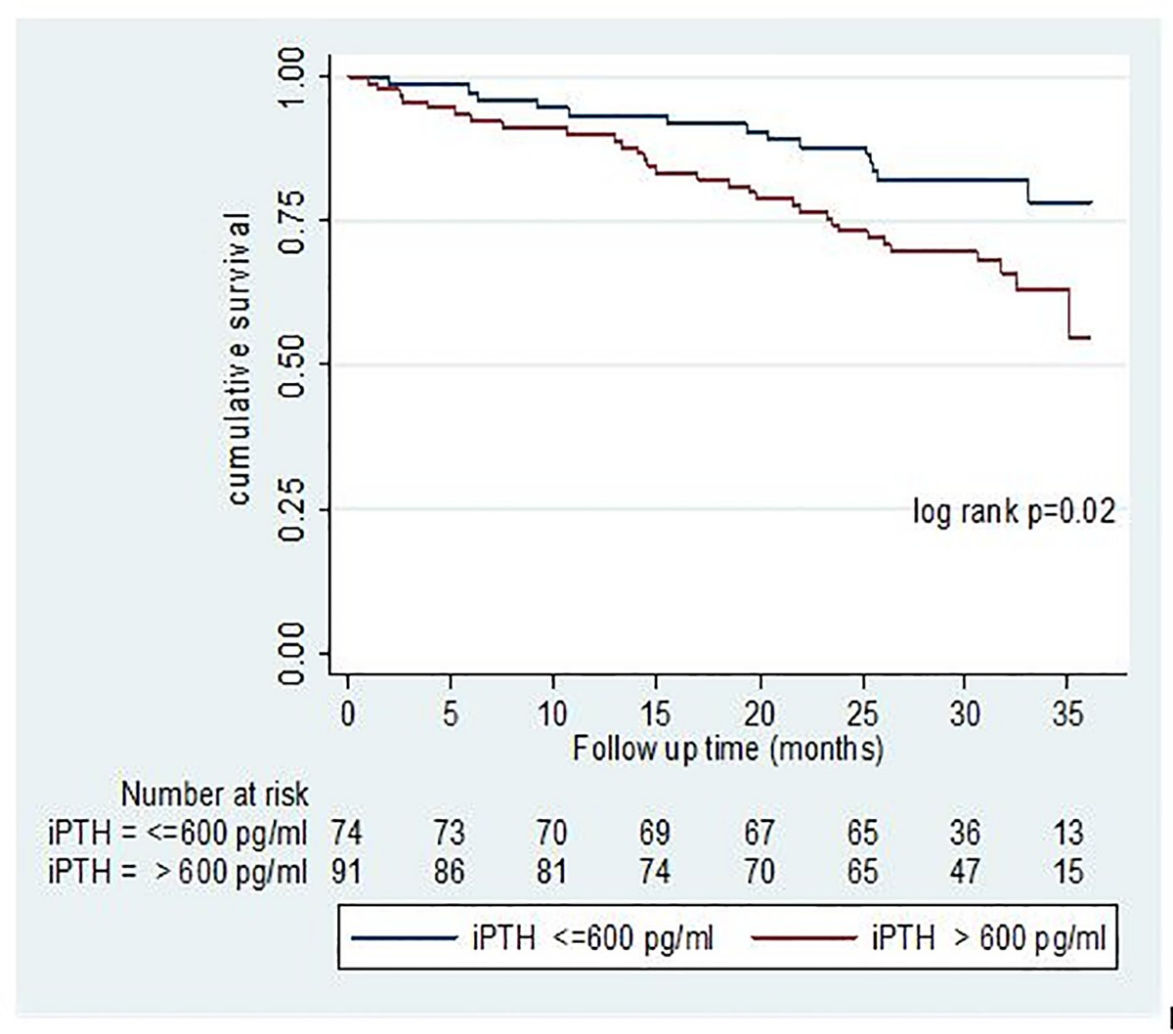
Kaplan Meier survival curves for categories of intact Parathyroid hormone.

The increased risk of death with higher levels of PTH remained unaltered when PTH was considered as a continuous exposure variable, HR per unit increased in log transformed PTH, 2.21; 95% CI, 1.07–4.55, P = 0.03 (Table 4). Fig 3 represents the Kaplan Meier survival curves by categories of intact parathyroid hormone.

**Fig 4:**
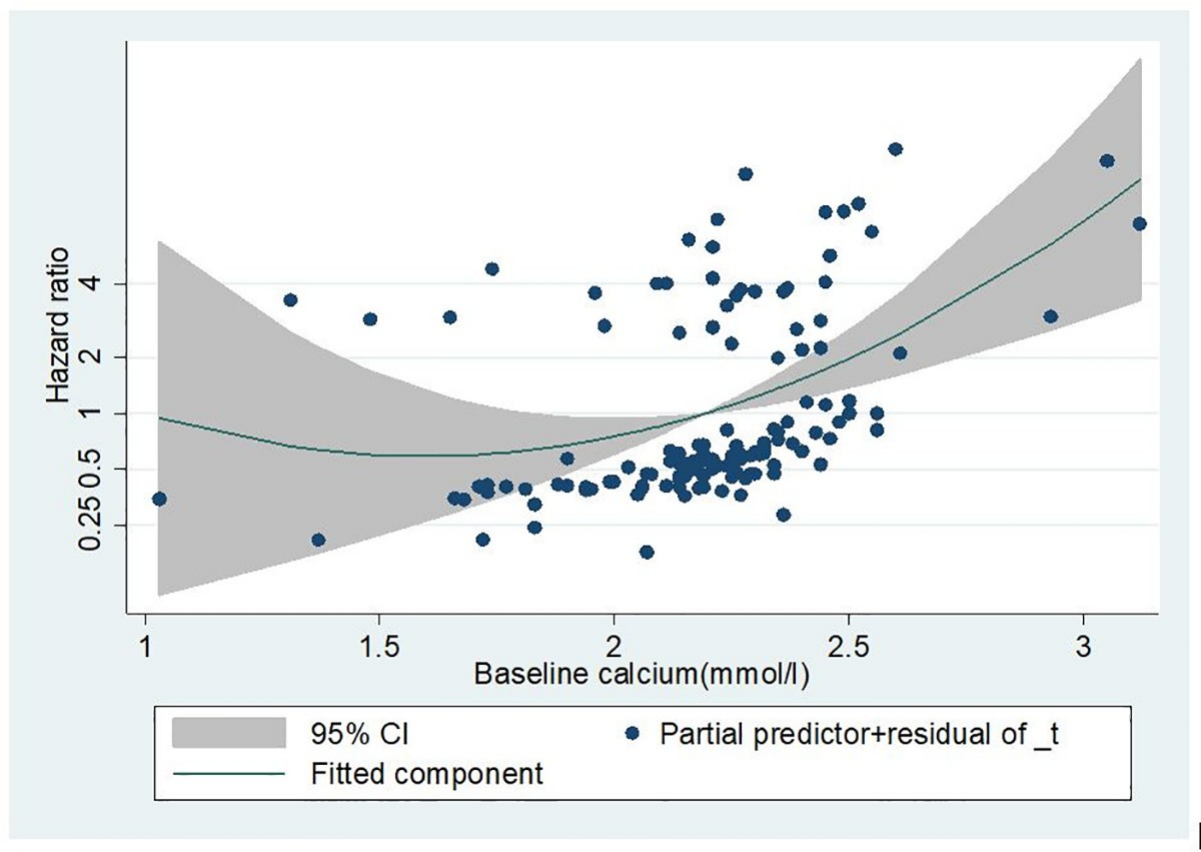
Hazard ratio of all- cause mortality for baseline serum calcium using fractional polynomial.

**Fig 5:**
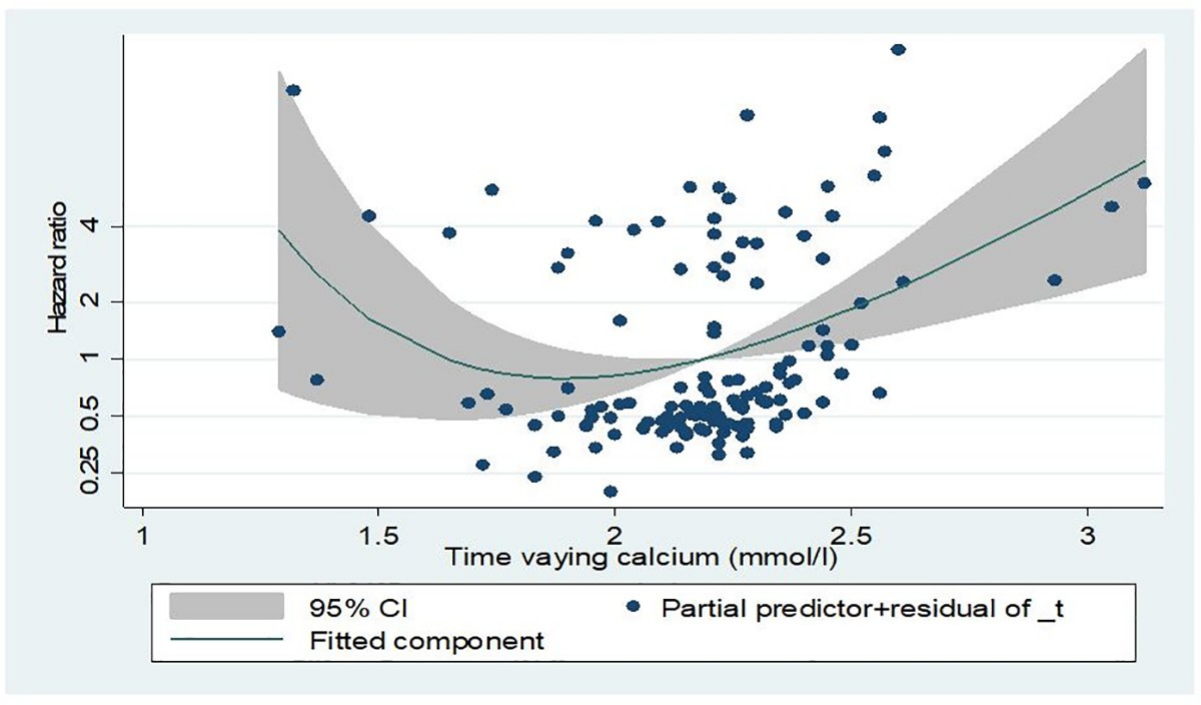
Hazard ratio of all-cause mortality for time dependent serum calcium using fractional polynomial.

Although the association between mortality and hypocalcaemia is not significant, the overall relationship seems to take a U- shaped pattern (Figs 4 and 5).

Similarly, when calcium is being considered as a time varying covariate, the increased risk of death with calcium remains unchanged (Table 3).

The significant association between serum calcium and all-cause mortality remains sustained even when serum calcium was treated as a continuous exposure variable [HR per unit increase in calcium; 5.34 (95% CI, 1.11–25.73), p = 0.04] (Table 4).

The fractional polynomial analysis furthers confirms this association and the U-shaped relationship (Figs 4 and 5)

### Phosphate and risk of mortality

In the baseline fixed model 1 that adjusted for markers of CKD–MBD, patients with high phosphate levels had increased risk of death compared to those with phosphate within the normal range, HR 2.35 (95% CI, 1.09–5.06; P = 0.03). This finding became attenuated and non-significant in the fully adjusted model 2, HR 1.64 (95% CI, 0.60–4.51; P = 0.33). This finding is also not consistent with time dependent analysis, which showed a non- significant association between higher levels of phosphate in both model 1 and model 2. However, when phosphate was analyzed as a continuous variable, increasing levels of phosphate were significantly associated with an increased risk of death in both model 1 [HR per unit increase in phosphate; 2.27 (95% CI, 1.11–4.64), P = 0.03] and model 2 [HR per unit increase in phosphate; 2.12 (95% CI, 1.02–4.42), P = 0.045] (Table 4).

### Bone specific alkaline phosphatase and mortality

In model 1 that adjusted for age and other biochemical markers of CKD-MBD, patients with BSALP > 15 IU/L versus 12–15 IU/L had a 2.91 fold significantly greater risk of death (HR 2.91, 95% CI, 1.12–7.54; P = 0.028). This significant association did not persist in the fully adjusted model (HR 2.12, 95% CI,0.77–5.87; P = 0.15).

### Race, FGF23 and mortality

In the fully adjusted model including an interaction between FGF23 and race, using black patients with FGF 23 lower than the overall median value as the reference, white participants with FGF 23 above the median value had similar HR with black patients with FGF23 above the median value (HR 3.24, 95% CI, 070–15.05; p interaction = 0.13 versus HR 3.28, 95% CI, 0.65–16.52; p interaction = 0.15).

## Discussion

In this prospective African study with diverse ethnicity (Blacks and Whites) on maintenance dialysis, higher levels of intact FGF23 levels did not seem to predict all-cause mortality. Conversely, higher levels of calcium and severe hyperparathyroidism were independently associated with all-cause mortality.

Previous studies relating to the association between FGF23 and clinical adverse outcomes have yielded conflicting results. Our findings of a lack of significant association between intact FGF23 levels was contrary to some previous studies [2, 14]. The discrepancy may partly be attributed to the use of different FGF23 assay methodologies and study populations. Although some of these studies similarly utilized intact FGF 23, it was reported that cardiovascular risk factors may be more specific with C- terminal FGF23[25]. For example, in a recent meta-analysis cumulative HR for c- terminal revealed a 2.3-fold increased risk of all-cause mortality, while higher levels of intact FGF23 were not significantly associated with mortality [13].

Although this meta-analysis excluded Gutierrez’s study in the pool analysis because odds ratios was reported as the measure of association with the outcome, the study utilized both intact FGF23 and C-terminal FGF23 assays revealing a strong linear correlation and similar findings with the two assays[2]. While some studies have shown a significant disagreement between the two assays[26, 27].

In addition to the use of different assay methodology, the impact of high levels of FGF23 also varied across races. For instance, in the same meta-analysis, no association was found between level of FGF23 and mortality in the Asian population (HR: 0.95; 95% CI: 0.37–2.43), while in non- Asian population elevated FGF 23 was significantly associated to mortality (HR: 1.69; 95% CI: 1.11–2.57) [13].

A similar trend in the variation of consequences of disordered markers of CKD -MBD across races was reported by the Multi-Ethnic Study of Atherosclerosis (MESA); in which 25-OHD deficiency was associated with an increased risk of coronary heart diseases in whites but not in blacks[28]. These contrasting findings may partly be explained by racial variations in the levels of FGF23 and some markers of CKD-MBD such as PTH, 25-OHD, and phosphate. For example, similar to this current study, other previous studies have shown that blacks have lower levels of FGF23 and higher PTH levels compared with whites[2, 11, 29].

The overall mechanisms proposed for the excess risk in mortality with elevated levels of C- terminal FGF23 are unclear. Some of the repeatedly documented mechanisms, though remaining speculative, include the direct cytotoxic effects of excess FGF23 on the myocardium leading to left ventricular hypertrophy, endothelial dysfunction, and arterial calcification [14, 15]. The controversy relating to these postulated modes of action was compounded by the absence of a coreceptor (Klotho) in the cardiovascular system which is needed by FGF23 to exert its effects on these tissues [30]. For example, in Chronic Renal Insufficiency study, Scialla et al reported that baseline FGF23 was not associated with arterial calcification, and also noted the absence of mRNA expression for FGF23 and Klotho in both human and mouse vascular tissue [30].

Although some researchers have proposed that the effect of FGF23 on the cardiovascular system is mediated through Klotho independent pathways [4, 31], the controversies relating to its mechanism prevail.

In agreement with our findings, previous studies have also reported that higher levels of FGF23 do not predict mortality in haemodialysis patients. Olauson et al, whose study population was similar to the present study comprising both haemodialysis and peritoneal dialysis patients, reported the lack of a significant association between higher levels of FGF23 and increased mortality risk, when FGF23 was analyzed in quartiles and on a continuous scale [8]. A similar trend was seen in a large cohort of patients with stage 3 CKD, where higher levels of FGF 23 failed to be associated with all-cause mortality and progression to CKD [9].

As we have previously published a survival advantage and a significant lower level of FGF 23 in our black CKD patients compared to white patients[12, 32], we further explored if there is an interaction between FGF23 levels, race and mortality risk. Similar to previous studies [14, 33], in this current study, black race did not modify the effect of FGF23 on mortality.

Our finding of a significant association between high levels of serum calcium and increased mortality risk is consistent with the findings of previous studies [16, 34]. Floege et al have also shown that hypercalcemia is a potent predictor of mortality in an European dialysis population and this finding was consistent when calcium was analyzed as fixed baseline and time varying covariates[16]. In line with our study, their findings were reinforced by modelling serum calcium as a continuous exposure variable using the fractional polynomial method. This limits loss of information associated with categorizing explanatory variables. Although cause and effect associations are difficult to be established with observational studies, the biological plausible explanation for the poor survival rate with high levels of calcium may be linked to the acceleration of arterial calcification by excess calcium.

Of note, we adjusted for the use of CKD-MBD medications (alfacalcidol and calcium carbonate) which have been associated with levels of serum calcium and phosphate, in addition to an inconclusive evidence that these medications may modify mortality in patients on haemodialysis[35].

Reports relating to PTH and mortality remain contradictory, while some studies have shown increased risk of death with elevated PTH, some have reported mixed results. The discrepancies between these studies may be related to different methodological issues, different cut off values used as reference points, study populations and practice patterns. For example, in a large European study comprising 8377 chronic haemodialysis participants, only intact PTH below the lower limit of KDIGO (<130pg/ml) target value was associated with lower survival, while PTH above the recommended upper limit (>585 pg/ml) was not predictive of mortality [36]. In contrast to this finding, several studies including this current study have shown an increased risk of death with PTH above the upper limit. In the DOPPS study Tentori et al. found that iPTH level >600 pg/mL was an independent predictor of mortality [37]. Similarly, Floege et al reported 2-fold increase in risk of death with PTH > 600 pg/ml [16]. The biological explanation for the strong association between PTH and mortality risk may likely be through a synergetic relationship with calcium. High levels of PTH will cause mobilization of excessive calcium from the bone which will in turn accelerate arterial calcification [6]. Moreover, the leading cause of mortality in dialysis patients has been repeatedly attributed to cardiovascular disease, with emerging evidence that arterial calcification may largely be responsible [6]; hence fulfilling some of the Brad Hill criteria of causation of an event.

However, despite the reported increased mortality risk with high levels of PTH, an available randomized controlled trial did not show a significant improvement in mortality with lowering PTH level. The trial titled “Evaluation of Cinacalcet Hydrochloride Therapy to Lower Cardiovascular Events (EVOLVE)” showed a non-significant 7% reduction in the risk of death with the use of cinacalcet to lower PTH level in patients with moderate to severe secondary hyperparathyroidism [38]. Thus, more randomized controlled trials are needed to ascertain the benefits of lowering PTH to recommended target values.

Another notable finding in this current study was the association between phosphate and increased risk of death when phosphate was factored into the model as a continuous variable, but not on categories. This further reiterates the fact that when exposure variables are analyzed only based on categories may erroneously lead to wrong conclusions. Hence, the need to further explore the relationship on a continuous scale as shown in this current study. This association is not surprising in the light of several studies that have consistently associated higher levels of phosphate with increased cardiovascular disease and all- cause mortality [34, 39]. This was reinforced by studies that reported improvement in survival with the use of phosphate binders to lower phosphate in patients with advanced CKD [40, 41]. Similarly, Sciella et al in an experimental study reported that elevated phosphate induced arterial calcification in vitro and that FGF23 played no role in the phosphate uptake during the process of the calcification [30].

Limitations of our study include relatively small sample size probably precluding detection of a significant association between higher levels of FGF 23 and mortality risk. However, as highlighted earlier, some studies with larger sample size with more events of interest also did not detect a significant association. FGF23 was measured at a single point in time, and since some exposures change over time, we were unable to model FGF23 as a time varying covariate. However, a recent study that assessed longitudinal FGF23 trajectories and risk of mortality in CKD patients showed that plasma FGF23 levels were stable over time in the majority of their study cohort [42]. Additionally, despite attempt to adjust for potential confounders, we still could not have accounted for other residual confounding variables. Finally, we could not ascertain specific causes of death and incidence of cardiovascular disease which is one of the leading causes of death in dialysis population. Meanwhile, the strengths our study include the diverse study population involving both black and white South African populations, and patients on both peritoneal and haemodialysis. Hence, allowing comparison of data not only between Black Africans and Black Americans but also between Whites in Africa and USA/Europe. In addition, previous studies relating to FGF23 were largely from US, Europe and Asia and thus this current study has given insights on the burden of FGF 23 and traditional markers of CKD-MBD in an African dialysis population.

Repeated measurements of the traditional markers of CKD- MBD have allowed us to account for variations of these markers on the impact of our primary endpoint over a period. Furthermore, modelling our explanatory variables on a continuous scale has further reinforced our derived findings based on categorical variables.

In conclusion, high levels of plasma PTH and serum calcium independently predicted all- cause mortality in diverse South African ESKD patients on dialysis, while FGF23 was not significantly associated with all- cause mortality in this cohort. These findings further reinforce the need to continuously pay attention in addressing traditional markers of CKD- MBD.

## Supporting information

S1 Table Participants’ characteristics by categories of intact Parathyroid hormone. (DOCX)

S2 Table Participants’ characteristics by categories of serum calcium. (DOCX)

S1 Data Anonymized data set. (DTA)
