# Supplementary material for: Associations of plasma fibroblast growth factor 23 and other markers of chronic kidney disease—Mineral and bone disorder with all-cause mortality in South African patients on maintenance dialysis: A 3-year prospective cohort study
Source: PLoS One. 2019 May 20;14(5):e0216656. doi: 10.1371/journal.pone.0216656 (PMC6527219; doi:10.1371/journal.pone.0216656)
Supplement: S1 Table — (DOCX) [file pone.0216656.s001.docx]

**S 1 Table Participants’ characteristics by categories of intact Parathyroid hormone**

|  |  | Intact PTH categories (pg/ml) | | |  |
| --- | --- | --- | --- | --- | --- |
| Variables | All=165 | <130 (n=29) | ≥130-≤600 (n=45) | >600 (n=91) | P Value |
| Age (years) | 46.6±14.2 | 42.0±15.6 | 50.6±16.1 | 45.7±12.2 | 0.03 |
| Gender n (%) |  |  |  |  |  |
| Male | 90 (54.5) | 18 (62.1) | 25 (55.6) | 47 (51.6) | 0.46 |
| Female | 75(45.5) | 11(37.9) | 20 (44.4) | 44 (48.4) |  |
| Race n (%) |  |  |  |  |  |
| Black | 111 (67.3) | 18 (62.1) | 22 (48.9) | 71 (78.00 | 0.001 |
| White | 54 (32.7) | 14(48.3) | 23 (51.1) | 48(52.7) |  |
| Dialysis Vintage (months) | 61(43-96) | 85(49-123) | 55(39-82) | 62(42-97) | 0.09 |
| DM status n(%) | 13 (7.9%) | 2(6.9) | 6 (1.3) | 5(5.5) | 0.29 |
| Hb (g/dl) | 10.8±2.1 | 10.6±2.3 | 11.0±2.2 | 10.7±2.1 | 0.66 |
| T.Cholesterol (mmol) | 4.30±1.43 | 3.74±1.10 | 4.08±1.27 | 4.50±1.52 | 0.08 |
| FGF23 (pg/ml) | 382(145-2977) | 244(101-1398) | 317(89-1566) | 716(184-3475) | 0.07 |
| Calcium (mmol/l) | 2.21±0.28 | 2.29±0.28 | 2.17±0.24 | 2.20±0.28 | 0.18 |
| Phosphate (mmol/l) | 1.52±0.55 | 1.52±0.51 | 1.39±0.54 | 1.59±0.56 | 0.15 |
| 25-OHD (ng/ml) | 27.7±13.6 | 30.8±12.9 | 26.1±14.8 | 27.5±13.6 | 0.39 |
| BSALP (U/L) | 15.8±5.5 | 16.2±4.6 | 16.1±5.4 | 15.5±5.5 | 0.79 |
| Albumin (g/L) | 36.1±6.6 | 39.6±6.0 | 35.1±5.5 | 36.1±6.0 | 0.55 |
| CKD MBD meds n (%) |  |  |  |  |  |
| Alfa calcidol | 104 (63.0) | 18 (62.1) | 25(55.6) | 60(65.9) | 0.38 |
| Calcium carbonate | 110 (66.7) | 18 (62.1) | 30 (66.7) | 62 (68.1) | 0.75 |

_T=Total; DM= diabetes mellitus; Hb= Haemoglobin; FGF23= Fibroblast growth factor; BSALP=Bone specific alkaline phosphatase; PTH=Parathyroid hormone, 25-OHD= 25 -hydroxyvitamin D; CKD-MBD-Chronic kidney disease- mineral bone disease, meds=medications._
