# Supplementary material for: Associations of plasma fibroblast growth factor 23 and other markers of chronic kidney disease—Mineral and bone disorder with all-cause mortality in South African patients on maintenance dialysis: A 3-year prospective cohort study
Source: PLoS One. 2019 May 20;14(5):e0216656. doi: 10.1371/journal.pone.0216656 (PMC6527219; doi:10.1371/journal.pone.0216656)
Supplement: S2 Table — (DOCX) [file pone.0216656.s002.docx]

**S2 Table Participants’ characteristics by categories of serum calcium**

|  |  | Calcium categories (mmol) | | | | |  |
| --- | --- | --- | --- | --- | --- | --- | --- |
| Variables | All=165 | <1.80 (n=13) | 1.80-2.10(n=24) | 2.11-2.37 (n=84) | 2.38-2.50 (n=24) | >2.50 (n=20 ) | p- value |
| Age (years) | 46.6±14.2 | 40.5±12.5 | 43.4±12.5 | 47.5±12.5 | 47.9±8.9 | 45.6±14.2 | 0.35 |
| Gender n (%) |  |  |  |  |  |  |  |
| Male | 90 (54.5) | 8(61.5) | 15(62.5) | 46(54.8) | 12(50.0) | 9(45.0) | 0.79 |
| Female | 75(45.5) | 5 (38.5) | 9(37.5) | 38(45.2) | 12(50.0) | 11(55.0) |  |
| Race n (%) |  |  |  |  |  |  |  |
| Black | 111 (67.3) | 12 (92.3) | 19(79.2) | 53(63.1 | 15(62.5) | 12(60.0) | 0.28 |
| White | 54 (32.7) | 1 (7.7) | 5 (20.8) | 31(36.9) | 9(37.5) | 8(40.0) |  |
| Dialysis Vintage (months) | 61(43-96) | 54(44-144) | 53(42-90) | 66(48-96) | 59(39-96) | 87(49-125) | 0.58 |
| DM status | 13 (7.9) | 1(7.7) | 1(4.2) | 7(8.3) | 3(12.5) | 1(4.2) | 0.69 |
| Hb (g/dl) |  | 9.9±2.2 | 10.5±2.0 | 11.0±2.3 | 10.3±1.7 | 11.4±2.2 | 0.26 |
| T.Cholesterol (mmol/l) | 4.30±1.43 | 4.47±1.46 | 4.26±1.49 | 4.31±1.46 | 4.53±1.17 | 3.73±1.18 | 0.70 |
| FGF23 (pg/ml) | 382 (145-2977) | 254(74-3881) | 244(122-1242) | 337(115-2977) | 1707(989-3475) | 603(103-36791) | 0.14 |
| PTH (pg/ml) | 750(268-1359) | 1097(805-1690) | 719(226-1331) | 689(297-986) | 1718(506-1889) | 129(32-1287) | 0.02 |
| Phosphate (mmol/l) | 1.52±0.55 | 1.65±0.54 | 1.42±0.39 | 1.51±0.54 | 1.53±0.43 | 1.56±0.51 | 0.79 |
| 25-OHD (ng/ml) | 27.7±13.6 | 23.4±11.4 | 24.7±10.0 | 27.1±13.2 | 33.5±17.6 | 32.0±16.2 | 0.09 |
| BSALP(U/L) | 15.8±5.5 | 16.6±7.0 | 16.1±4.4 | 15.5±5.4 | 16.4±4.4 | 15.3±3.2 | 0.79 |
| Albumin (g/dl) | 36.6±6.6 | 33.6±8.7 | 36.2±7.2 | 35.9±5.4 | 38.2±5.9 | 39.2±4.9 | 0.12 |
| CKD-MBD meds n (%) |  |  |  |  |  |  |  |
| Alfacalcidol | 104(63.0) | 10 (76.9) | 16(66.7) | 49(58.3) | 15(62.5) | 14(58.3) | 0.65 |
| Calcium carbonate | 110(66.7) | 11(84.6) | 19(79.2) | 50(59.5) | 17(70.8) | 13(54.2) | 0.06 |

_T=Total; DM= diabetes mellitus;; Hb= Haemoglobin; FGF23= Fibroblast growth factor; BSALP=Bone specific alkaline phosphatase; PTH=Parathyroid hormone, 25-OHD= 25 -hydroxyvitamin D; CKD-MBD-Chronic kidney disease- mineral bone disease, meds= medications_
